# Full Sequence and Comparative Analysis of the Plasmid pAPEC-1 of Avian Pathogenic *E. coli* χ7122 (O78∶K80∶H9)

**DOI:** 10.1371/journal.pone.0004232

**Published:** 2009-01-21

**Authors:** Melha Mellata, Jeffrey W. Touchman, Roy Curtiss

**Affiliations:** 1 The Biodesign Institute, Arizona State University, Tempe, Arizona, United States of America; 2 School of Life Sciences, Arizona State University, Tempe, Arizona, United States of America; University of Liverpool, United Kingdom

## Abstract

**Background:**

Extra-intestinal pathogenic *E. coli* (ExPEC), including Avian Pathogenic *E. coli* (APEC), are very diverse. They cause a complex of diseases in Human, animals, and birds. Even though large plasmids are often associated with the virulence of ExPEC, their characterization is still in its infancy.

**Methodology/Principal Findings:**

We fully sequenced and analyzed the large plasmid pAPEC-1 (103,275-bp) associated with the APEC strain χ7122, from worldwide serogroup O78∶K80∶H9. A putative virulence region spanning an 80-kb region of pAPEC-1 possesses four iron acquisition systems (*iutA iucABCD*, *sitABCD*, *iroBCDN*, and temperature-sensitive hemagglutinin *tsh*), a colicin V operon, increasing serum sensitivity *iss*, *ompT*, *hlyF*, and *etsABC*. Thirty three ORFs in pAPEC-1 are identified as insertion sequences (ISs) that belong to nine families with diverse origins. The full length of the transfer region in pAPEC-1 (11 kb) is shorter compared to the *tra* region of other sequenced F plasmids; the absence of some *tra* genes in pAPEC-1 affects its self-transferability, and the conjugative function of the plasmid was effective only in the presence of other plasmids. Two-replicon systems, *repFIIA-repFIC* and *repFIB*, and two post-segregational systems, *srnB* and *hok/sok*, are also present in the sequence of pAPEC-1. The comparison of the pAPEC-1 sequence with the two available plasmid sequences reveals more gene loss and reorganization than previously appreciated. The presence of pAPEC-1-associated genes is assessed in human ExPEC by PCR. Many patterns of association between genes are found.

**Conclusions/Significance:**

The pathotype typical of pAPEC-1 was present in some human strains, which indicates a horizontal transfer between strains and the zoonotic risk of APEC strains. ColV plasmids could have common virulence genes that could be acquired by transposition, without sharing genes of plasmid function.

## Introduction

Plasmids are influential factors in the pathogenesis and evolution of bacteria [1]. Their ability to transfer between species is a way for plasmids to acquire new genes or target new hosts. Characterizing plasmids from different bacteria is a key pursuit towards understanding the mechanism of virulence and their evolution, and the design of efficacious strategies to fight diseases.

Avian pathogenic *E. coli* (APEC) strains cause a complex of diseases in birds, mainly respiratory [2], [3]; and because their phylogenic background, APEC are also suspected to be a potential zoonotic risk for human [4], [5]. APEC is a subgroup of the extra-intestinal pathogenic *E. coli* (ExPEC) pathotype [6], which includes uropathogenic *E. coli* (UPEC), neonatal-meningitis *E. coli* (NMEC), and septicemic *E. coli*. APEC strains are very diverse, and their diversity is related to the diversity of their virulence factors and serotypes. They belong mostly to the serogroups O1, O2, and O78, or are often nontypable. The pathogenicity of APEC is still poorly understood. Similar to other ExPEC members, APEC strains possess virulence factors that allow them to have a systemic life. The most prevalent virulence traits associated with APEC are adhesins (type 1 and P fimbriae, Temperature-sensitive hemagglutinin Tsh, and curli), capsules, iron acquisition systems and serum resistance [2], [3].

Large plasmids are commonly associated with virulence of APEC. Their importance has been demonstrated by the occurrence of plasmid-encoded genes shared among APEC strains [7] and the presence of virulence genes in plasmids [8], [9]. According to recent studies, most virulence genes associated with APEC are often located on IncF plasmids, named ColV plasmids because their ability to code for production of colicin V, a small protein from the microcine family [8], [10], [11]. ColV plasmids are associated with *E. coli* in general and with APEC in particular. Their link with the ability of bacteria to cause disease in production animals has been demonstrated [12]–[14]. *In vivo* expression of genes encoded by the ColV plasmid, including Tsh, aerobactin, and the Iro system, has been confirmed by using SCOTS [7] and the occurrence of the putative virulence genes of APEC on non-ColV plasmids has also been shown [7], [15], [16].

Although large plasmids are commonly found in APEC strains [16]–[18] and are often considered a feature of the APEC pathotype [18], only a few of them, mainly from serogroups O1 and O2, have been sequenced [15], [19]. Our previous studies investigated the involvement of a large plasmid, pAPEC-1, in the virulence of APEC strain χ7122 from a worldwide serogroup O78∶K80∶H9 [16]. We showed that pAPEC-1-cured χ7122 bacteria were attenuated in chickens, caused few lesions of pericarditis and perihepatitis, did not persist in the blood, and poorly colonized the lung, spleen, and liver [16]. Our results also demonstrated a decrease in the resistance of strains to the bactericidal effects of heterophils and macrophages *in vitro* [20]. Even though pAPEC-1-cured bacteria were still resistant to serum [16], [21]; the presence of pAPEC-1 in the K-12 strain increased its ability to survive in serum by about 100-fold [16]. Herein, we (i) present and analyze the full sequence of the plasmid pAPEC-1 of APEC strain χ7122 (O78∶K80∶H9), (ii) investigated the pAPEC-1 conjugal transfer mechanism and (iii) determine the prevalence of pAPEC-1-associated virulence genes in human ExPEC and their phylogenetic relationship.

## Materials and Methods

### Strains, plasmids, media, mating and growth conditions

Bacterial strains and plasmids used in this study are listed in Table 1. A collection of one hundred human strains isolated from the main clinical extra-intestinal sources (UTI and non-UTI), kindly provided by Dr. James R. Johnson (VA Medical Center, Uni. Minnesota), were used to study the distribution of pAPEC-1-associated genes in human ExPEC using the PCR.

**Table 1:** Bacterial strains and plasmids used in this study.

Strain/ plasmid	Relevant characteristics*	Reference or source
**Strains**		
MGN-617	SM10 λ*pir* derivative, *thi thr leu tonA lacY supE λpir recA*::RP4-2-Tc::Mu (Km^r^) Δ*asdA1*	[33]
TOP10	*E. coli* K-12, Lac^−^ F^−^ Str^r^	Invitrogen
χ833	*E. coli* K-12, Lac^−^ F^−^ Str^r^	This study
χ2934	*E. coli* K-12, Lac^−^ F^−^ Nal^r^	This study
χ6092	*E. coli* K-12, Lac^−^ F^−^ Tc^r^	This study
χ7122	APEC O78∶K80∶H9, *gyrA* Nal^r^	[22]
χ7273	χ7122 *tsh*::*tetAR*(B), Nal^r^ Tc^r^	[16]
χ7276	*E. coli* K-12 MG1655 Tn*10*::*kan*, Km^r^	[16]
χ7277	χ7276 pAPEC-1-1 pAPEC-2, Km^r^ Tc^r^	[16]
χ7345	χ2934 pAPEC-1-1 Nal^r^ Tc^r^	This study
χ7346	χ6092 pAPEC-1 Tc^r^	This study
χ7396	MGN-617 pAPEC-1-1, Km^r^ Tc^r^ Asd^−^	This study
39R681	*E. coli* containing four plasmids 147 kb, 63 kb, 35.85 kb, and 6.9 kb	[83]
**Plasmids**		
pAPEC-1	Virulence plasmid of APEC χ7122	[16]
pAPEC-1-1	pAPEC-1 *tsh*::*tetAR*	[16]

* Km, kanamycin; Nal, nalidixic acid; Str, streptomycin; Tc, tetracyclin.

**Figure 1:**
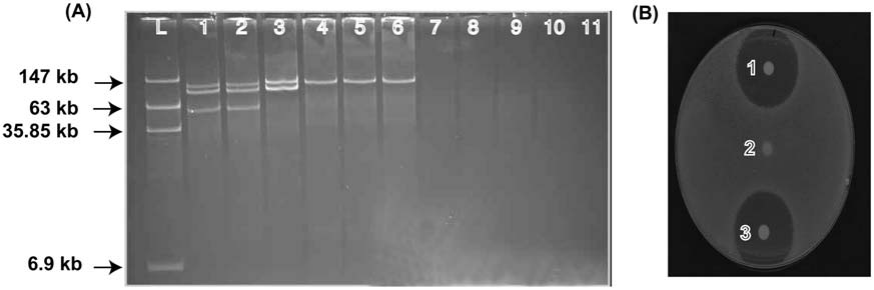
Plasmid profiles of strains on 0.5% agarose gel (A). Four plasmids (147 kb, 63 kb, 35.85 kb, and 6.9 kb) of the strain 39R681 [83] were used as a ladder (L). Each lane contained plasmids from the following bacterial strains: (1) χ7122, (2) χ7273, (3) χ7277, (4) 7345, (5) 7346, (6) χ7396, (7) χ833, (8) χ2934, (9) χ6092, (10) MGN-617, and (11) TOP10; Colicin production of strains (B). (1) χ7122, (2) χ6092, (3) χ7346.

APEC strain χ7122 (O78∶K80∶H9), isolated from liver of a deceased Turkey [22], possesses three large plasmids [[16], Fig. 1]; the largest of these three plasmids is termed pAPEC-1 and is considered to be the virulence plasmid of χ7122 [16]. The APEC strain χ7122 (Nal^r^) was mated with an *E. coli* K-12 χ6092 (Tc^r^), an avirulent strain lacking plasmids. The transconjugant χ7346 was selected on MacConkey agar containing tetracycline and colicin V synthesized in vitro from the χ7122 as described before [23].

For practical reasons, we used a plasmid with a tetracycline resistance marker pAPEC-1-1 (Table 1) to test the self-transferability of the plasmid. The strain χ7277 (Km^r^, Tc^r^) was mated with *E. coli* K-12 χ2934 (Nal^r^) to generate the strain χ7345. pAPEC-1-1 DNA purified from χ7345 was transferred by electroporation as previously described [24] into *E. coli* K-12 MGN-617 to generate the strain χ7396 (Table 1). The strains χ7345 and χ7396 containing pAPEC-1-1 were then mated with different plasmidless recipient strains χ833, χ2934, and TOP10 (Table 1). The contra selection was made on MacConkey agar with appropriate antibiotics.

The presence of plasmids in different bacterial strains was verified by agarose gel (0.5%) electrophoresis by the method of Kado and Liu [25]. Colicin production was determined by the double-layer method as described previously [26].

Strains were stocked at −80°C in 25% glycerol. LB broth and MacConkey agar supplemented with 1% lactose were used to grow bacterial strains. Antibiotics were added, as required, at the following concentrations: kanamycin, 30 µg/ml, nalidixic acid, 15 µg/ml; streptomycin 50 µg/ml, and tetracycline, 10 µg/ml. DAP was added (50 µg/ml) for the growth of Asd^−^ strains.

### PCR conditions

The size of the *traI-traB* region of pAPEC-1 was verified by PCR as previously described [16] using primers TraI F: 5′-TCAGTCTCCGCCCAGGGTTTTCTCTTTC-3′ and TraB R: 5′- ATGGCCAGTATCAATACCATTGTGAAAC-3′. The primers for genes *tsh*, *iss*, *cvaC*, *iroN*, *iucC*, *sitA*, *ompT*, *hlyF*, and *etsA* were designed from the pAPEC-1 sequence by using the Primer Premier 5.0 program (Table S1). PCR reactions were repeated at least three times; χ7122 and χ6092 were used as positive and negative controls respectively. The PCR products were purified from the agarose gel by using Qiaquick Gel Extraction Kit (Qiagen) and sequenced by the TGen DNA Sequencing Core (http://www.tgen.org).

### DNA purification

The pAPEC-1 and pAPEC-1-1 plasmid DNAs were purified from an overnight culture of transconjugants χ7346 and χ7396 respectively, grown in Luria-Bertani (LB) broth, by using a Large-construct Kit (Qiagen), according to the manufacture's instructions.

### Plasmid DNA sequencing

Purified pAPEC-1 plasmid DNA was fragmented by kinetic shearing, and a shotgun library was generated in the vector pOTWI3 using a size fraction of 3–5 kb. The pAPEC-1 sequence was established from 1,052 end sequences from the pOTWI3 library (achieving a 7.9× sequence coverage) using dye-terminator chemistry on ABI 3730xl automated sequencers. The sequence was assembled with the program Phrap (www.phrap.org) and finished as described previously [27]. Assembly accuracy was confirmed by forward and reverse read-pair concordance of individual plasmid subclones.

### DNA analysis

Initial automated annotation of the genome was performed with the TIGR/JCVI Annotation Engine (www.tigr.org/AnnotationEngine), where it was processed by TIGR's prokaryotic annotation pipeline. The manual annotation tool Manatee (manatee.sourceforge.net) was used to carefully review and confirm the annotation of every gene. Pseudogenes contained one or more mutations that would ablate expression; each inactivating mutation was subsequently checked against the original sequencing data. Truncations represent genes that are missing the 5′ or 3′ end of the coding sequence determined by protein sequence comparisons with orthologous sequences from other species. The Vector NTI suite of programs was used to confirm the previous analysis. Insertion sequences (IS) elements were identified by comparison with the IS database www-is.biotoul.fr.

Phylogenetic trees were generated by comparing the amino acid sequence of nine pAPEC-1-associated virulence genes with those of related sequences of other bacteria obtained from the GenBank database by neighbor-joining (1,000 replicates) using molecular evolutionary genetics analysis software version 4.0 (MEGA4) (http://megasoftware.net/). Bootstrap values are indicated at branch positions.

### Genomic comparison

Pairwise nucleotide comparison of the complete pAPEC-1 DNA sequence to that of pAPEC-O1-ColBM (NC_009837) and pAPEC-O2-ColV (NC_007675) were generated with the Artemis comparison tool, webACT (http://www.webact.org/WebACT). Same-strand DNA similarity is shaded red, while reverse similarity is shaded blue. Aligned regions greater than 1000 base pairs with a percent-identity >96% are shown. The default BLASTn parameters were used (low complexity regions were masked; nucleotide mismatch penalty: -3; reward for nucleotide match: 1). The circular genome map was created using the program CGView [28]. The complete and annotated genome sequences of pAPEC-1 have been deposited in the DDBJ/EMBL/GenBank database under accession number CP000836.

## Results and Discussion

### General overview of the plasmid pAPEC-1

**Figure 2:**
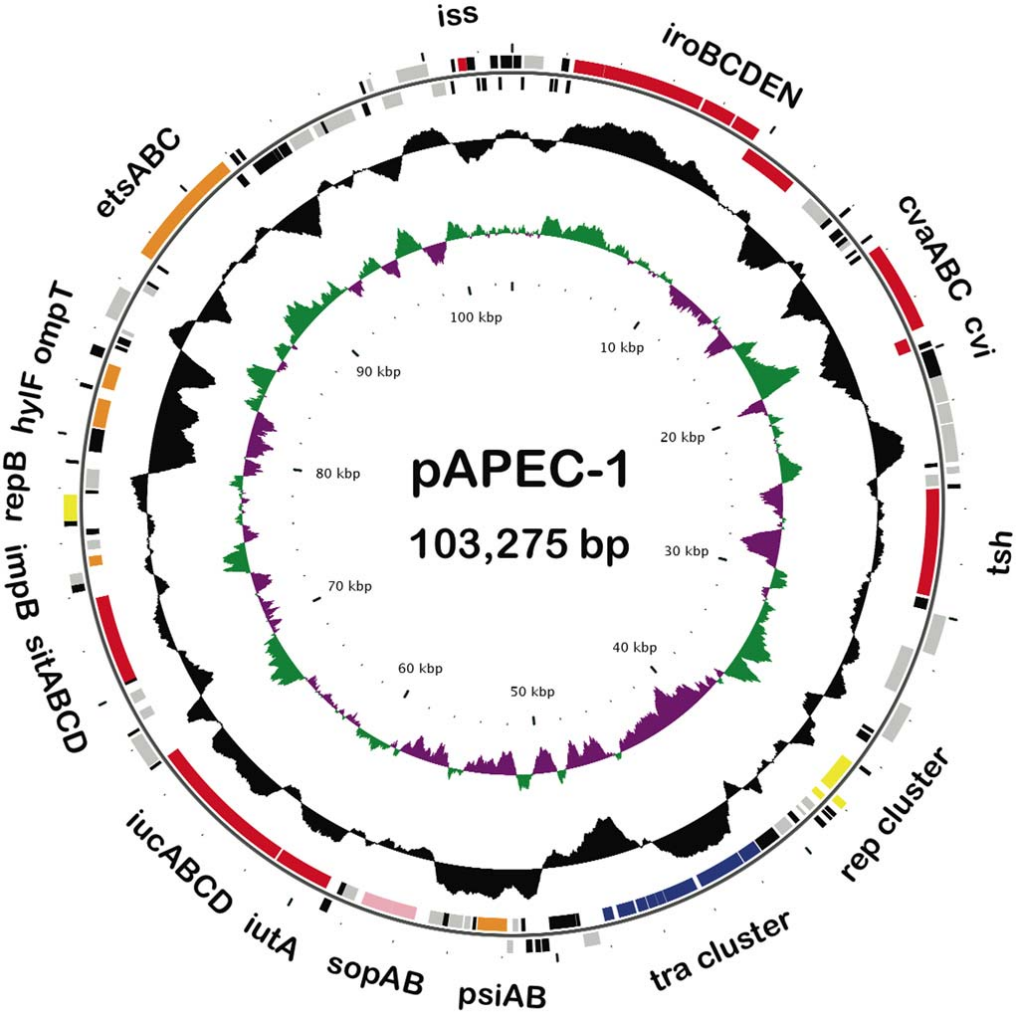
Circular representation of the *E. coli* pAPEC-1 plasmid. The different rings represent (from outer to inner) all genes and insertion elements, which are color coded by functional group (rings 1 and 2), deviation from average G+C content (ring 3), and GC skew ((G−C)/(G+C); ring 4). Colors represent the following: red, virulence associated; green, mobile elements; blue, plasmid transfer; yellow, plasmid replication; orange, unknown; pink, plasmid maintenance; black, hypothetical proteins; and gray, other functions.

The entire nucleotide sequence of the first large plasmid, pAPEC-1, of APEC strain χ7122 (O78∶K80∶H9) was determined. pAPEC-1, an IncF colicin V plasmid [[16], Fig. 1] consists of 103,275 base pairs forming a circular plasmid (Fig. 2) with an average GC content of 48.7%, slightly lower than the genome of *E. coli* (50.8%) [29], the G+C content fluctuates along the pAPEC-1 sequence (Fig. 2). Detailed analysis of the plasmid sequence predicted the presence of 163 open reading frames (ORFs) and 3 pseudogenes as determined by automated annotation (see Methods) (Table S2). The distribution of the pAPEC-1 ORF start codon usage is 67.5% ATG, 19.9% GTG and 12.7% TTG. Of the 163 ORFs, 31 genes (19%) encode proteins similar to known and putative virulence genes (Table S2), 26 (15.95%) encode proteins involved in plasmid functions (Table S3), 33 (20.24%) are similar to insertion sequence genes (Table S4), 27 (16.6%) are predicted proteins conserved in other species, and 46 genes (28.22%) are predicted proteins with no similarity to proteins in public databases.

### Maintenance and replication systems

A complete list of DNA sequences involved in pAPEC-1 functions are presented in Table S3, however, details of most interesting traits within this region are discussed below.

### Transfer (*tra*) region of pAPEC-1 is truncated

**Figure 3:**
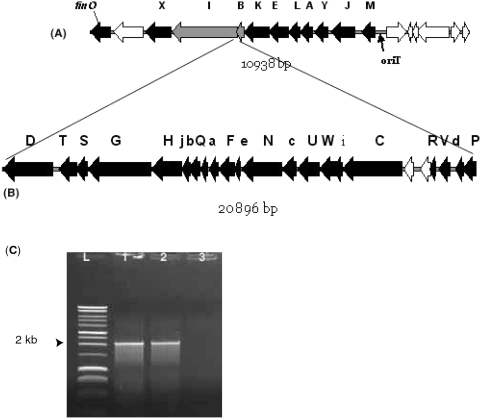
Genetic map of the transfer region of pAPEC-1 (A), ORFs of pAPEC-O2-ColV absent in pAPEC-1 (B). Capital letters represent *tra* genes and lower case letters represent *trb* genes. Black arrows represent known ORFs genes, grey arrows represent truncated genes, and white arrows represent hypothetical protein genes. PCR of the *traI-traB* region on 0.8% agarose gel (C), (L) 10 kb ladder (Promega), (1) χ7122, (2) χ7346, (3) χ6092.

Conjugative plasmids in gram-negative bacteria possess a transfer region that encodes genes of the T4SS family responsible for the horizontal transfer of plasmids between organisms [30]. All of the ORFs assigned to putative transfer conjugative functions are located within an 11 kb region of the pAPEC-1 plasmid, referred to as the *tra* cluster (Fig. 2). Similar to other F plasmids, the *tra* region in pAPEC-1 is boarded by the origin of transfer (*oriT*) and the fertility inhibition gene, *finO* [[31]; Fig. 3A]. The full length of the transfer region in pAPEC-1 is shorter when compared to the *tra* regions of other sequenced F plasmids [31], [32], including avian plasmids pAPEC-O1-ColBM [19] and pAPEC-O2-ColV [15]. A segment of 23 ORFs from pAPEC-O2-ColV located between *traI* and *traB* is absent from pAPEC-1 (Fig. 3B). In fact, the size of the *traI-traB* region of pAPEC-1, around 2 kb as determined by PCR (Fig. 3C), confirmed the deletion of this large segment of DNA between these two genes.

Overall, the 10 genes within the *tra* region in pAPEC-1 plasmid are more similar at the nucleotide level (99 to 100% similarity) to their counterparts in pAPEC-O1-ColBM than to pAPEC-O2-ColV. In fact, *traY* and *traJ* in pAPEC-1 have no similarity in pAPEC-O2-ColV, whereas *traM* has only 85% similarity. The genes *traI* (1,884 bp) and *traB* (222 bp) of pAPEC-1 are truncated. The absence of 1482 bp from the C-terminal region of the *traI* gene and 1268 bp from the N-terminus of the *traB* gene is probably due to the deletion of the *tra* region containing 23 ORFs located between the two genes (Fig. 3). Besides the *tra* genes, pAPEC-1 possesses the two conserved genes among a range of transmissible plasmids, *ssb* and *psiB* (Fig. S1A). These genes are predicted to contribute to the conjugative processing of plasmids [31].

### Mechanism of pAPEC-1 conjugal transfer

We previously demonstrated that pAPEC-1-1 could be transferable from derivative APEC strain χ7273 as a donor to an *E. coli* K12 via mapping [16]. However, the full sequence of the plasmid revealed that some genes encoding conjugative function are missing (Fig. 3). To determine the mechanism of plasmid conjugal transfer, we tested the ability of pAPEC-1-1 to transfer from different donors. Our results showed that pAPEC-1 and pAPEC-1-1 were conjugated from the donor strains including χ7122, χ7273, χ7277 and χ7396 into different *E. coli* K-12 recipient strains (Table 1, Fig. 1), whereas the plasmid pAPEC-1-1 was not conjugated from the donor χ7345 into different recipient strains.

All together, our results suggest that the conjugative transfer function of pAPEC-1 seems to be effective only in the presence of other large plasmids included pAPEC-2 and pAPEC-3 in χ7122 and χ7273, or pAPEC-2 in χ7277, but also in universal donor strain MGN-617 [33], which has the *tra* region within the bacterial chromosome; the inability of pAPEC-1-1 to conjugate from χ7345, that contains pAPEC-1-1 alone (Table 1, Fig. 1), into different recipient *E. coli* strains confirms these results. The genes in the *tra* region that are missing in pAPEC-1 include genes involved in pilus assembly (*traV*, *traU*, *traF*, *traW*, *traC*, *traH*, *traQ*, *and trbC*), in control DNA transfer (*traD*), surface exclusion (*traT* and *traS*), and others (*traR*, *trbG*, *trbH*, *trbF*, *trbD*, and *trbI*) [31] affected the self-transferability of pAPEC-1 and rendered it depend on the transfer machinery of other conjugative plasmids present in the recipient strain. The sizes of plasmid were similar in both donor and recipient (Fig. 1) indicating that no plasmid recombination occurred; in addition, the absence of other plasmids with pAPEC-1-1 in the recipient strains suggests that resolution of co-integrate structures did not occur. The pAPEC-1 plasmid could only use the transfer machinery of other plasmids to compensate for its defective apparatus without necessarily co-integrated to the helper plasmids.

### Replication (rep) regions of ColV plasmids are not necessarily the same

**Figure 4:**
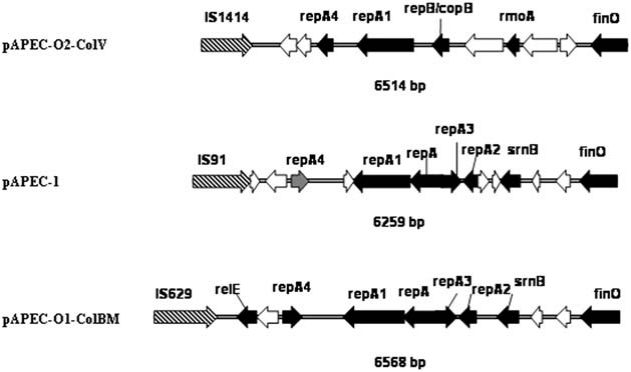
Comparison of the genetic map of the replication region of pAPEC-1 to that of pAPEC-O2-ColV (NC_007675) and pAPEC-O1-ColBM (NC_009837). Black arrows represent known ORFs genes, grey arrows represent truncated genes, and white arrows represent hypothetical protein genes and hatched arrows are Insertion Sequences genes.

Many plasmids of the IncF group have been shown to possess more than one replicon [15], [19], [34], [35]. Two-replicon systems are identified in the sequence of pAPEC-1. Of interest, the organization of the first replicon *repFIIA-repFIC*, located upstream of the transfer region, is more similar to the replicon identified on pAPEC-O1-ColBM [19] than to the homologue on pAPEC-O2-ColV [15] (Fig. 4), which suggests that ColV plasmids are not necessarily from the same backbone. The particularity of the pAPEC-1 transfer region is the presence of *repA*, a component of *repFIC* in the *repFIIA* region (Fig. 4) and an ORF that has similarity with sequence of pAPEC-O1-ColBM only. Genes of this region are identical (at the nucleotide and protein levels) to the genes of the *repFIIA* region in pAPEC-O1-ColBM. In pAPEC-1, with the exception of the *repA4* that has the lowest homology (93%) and contains a punctuation mutation (G-T) that creates the stop codon TGA in position 88. Two ORFs upstream of *repA4* do not have any homology with either pAPEC-O1-ColBM or pAPEC-O2-ColV plasmid sequences. The replication regions of the three sequenced avian plasmids pAPEC-O2-ColV, pAPEC-1, and pAPEC-O1-ColBM are flanked by different insertion sequences, IS*1414*, IS*91*, and IS*629* respectively (Fig. 4), which demonstrates that these regions could have different origins.

The second replicon found in pAPEC-1 is like *repFIB* according to sequence similarity (Fig. S1B). It is located downstream of the transfer region between the *sitABCD* and *etsABC* regions (Fig. 2). This replicon is homologous to the RepFIB relicons of pAPEC-O2-ColV [15] and pAPEC-O1-ColBM [19].

### Partition system (*sopABC*)

To ensure the fidelity of transmission of genes among cells of a population, low copy plasmids possess partitioning (*par*) loci, a mitotic process encoding for an active partition system governed by a set of three genes *sopABC* or *parABS* [36]. pAPEC-1 has a complete F plasmid *par* locus (*sopA*, *sopB*, and *sopC*) (Fig. S1A) located downstream of the *tra* region (Fig. 2); this locus is highly similar to the sequence in pAPEC-O1-ColBM but is absent in pAPEC-O2-ColV. pAPEC-1 contains also a truncated *parB* of the *parAB* system and lacks *parA*. The 5′ end of *parB* in pAPEC-1 was found to be fused with an IS*1* element (Fig. S1B). The possession of two Par modules by some plasmids would provide a means of avoiding competition with plasmids containing only one homologous set [37].

### Post-segregational system (toxin-antitoxin)

Two post-segregational systems, *srnB* and *hok/sok*, were identified in the pAPEC-1 sequence; they are located upstream and downstream of the *tra* region, respectively. The presence of two toxin-antitoxin systems on the same plasmid is not unusual; a similar situation is found with the pAPEC-O1-ColBM plasmid [19]. Different systems, including *hok/sok* of R1, *srnB* of F and *pnd* of R483, mediate maintenance by killing of plasmid-free segregants: The systems encode highly stable toxins (Hok, SrnB and PndA) that kill the cells from within [38], [39]. Their expression is repressed by the action of small unstable antisense RNAs that are complementary to the toxin mRNAs. The two systems from R and F plasmids suggest that pAPEC-1 may be the result of recombination between R and F plasmids.

### Plasmid-encoded functions in pAPEC-1

The putative virulence region of pAPEC-1 spans approximately 80 kb. This region contains four iron acquisition systems (*iutA iucABCD*, *sitABCD*, *iroBCDN* and temperature-sensitive hemagglutinin *tsh*), colicin V operon, increasing serum sensitivity *iss*, *ompT*, *hlyF*, and *etsABC* (Fig. 2). An analysis and discussion of four regions are presented below.

### ABC transport systems

The sequence of pAPEC-1 revealed the presence of at least five known ABC transport systems, including three iron acquisition systems (*iroBCDN*, *sitABCD*, and *iutA iucABCD*), a colicin V operon (*cvi cvaABC*), and a recently identified ABC transport (*etsABC*) [15] (Fig. 2). ABC transporters are integral membrane proteins that carry diverse substrates across lipid bilayers [40]. In bacteria, ABC transporters have a diverse range of functions that may be required for survival in different niches in response to the environments in which bacteria live. They catalyze the uptake of essential nutrients (sugars, amino acids, peptides, metal ions, iron) or the extrusion of toxic substances, thus contributing to drug and antibiotic resistance of microbial pathogens [41].

Iron is an essential cofactor of many enzymes and bacteria require iron concentrations at around 10^−7^ to 10^−5^ M to achieve optimal growth. The level of free iron is estimated to be very low in the environment and biological fluids (10^−18^ M) [42]. Only bacteria that have strategies to acquire iron sequestered by the host can survive in specific niches and consequently cause infections. The SitABCD was previously identified in APEC strain χ7122 and its role in transport of manganese and iron has been demonstrated [43]. The *sit* operon is multifunctional; it acts as an iron acquisition system during intracellular infection and as a transporter of different compounds, such as manganese, at different stages of infection [43], [44]. The role of SitABCD or their homologues in the virulence of *Salmonella*, *Shigella*, *Yersinia* and APEC has already been determined [45]–[48]. SitABCD has a role mainly in macrophages where nutrient levels are low and the level of oxidants is potentially high.

The siderophore, salmocheline *iroABCDN*, was first detected in *Salmonella* Typhi and later in other *Salmonella* strains as well in septicemic *E. coli* [49]–[51]. IroB and IroD have a glycosyltransferase and an esterification function, respectively [52]. Aerobactin, another iron acquisition system that consists of five genes *iucABCD* and *iutA*, was originally found to be encoded in the pColV-K30 plasmid [53], [54], but has also been found to be encoded in chromosomal genes of invasive bacteria [55]. The *iro* genes and aerobactin-encoding systems play a role in persistence and generation of lesions by APEC strain χ7122 in chickens and they function in concert to increase bacterial acquisition of iron within internal organs [7]. According to Valdebenito *et al.* [56], environmental factors, including pH, temperature and source of sugar influence the production of different iron acquisition systems present in *E. coli* strains suggesting that bacteria may need multiple iron acquisition systems to adapt to environment changes during host infection.

The two divergent operons of colicin V production are present in pAPEC-1 (Fig. 2) and are functional (Fig. 1). They consist of genes for ColV synthesis (*cvaC*) and ColV immunity (*cvi*) and two genes for ColV export (*cvaA* and *cvaB*) [57]. The operon ColV in pAPEC-1 is highly similar at the sequence level (99% identity) to an operon found in the plasmid pAPEC-O2-ColV [15]. The colicin V gene, identified for the first time in *E. coli* V by Gratia in 1925 [58], is a microcin produced by *E. coli* and acts against phylogenically related microbial strains by disrupting their cell membrane potential [59]. The colicin V is exported by CvaA and CvaB into the cytoplasmic membrane and outer membrane by a host chromosomal gene product TolC [57]. Colicin V provides a competitive advantage in colonization of the intestinal tract [60]. Many studies have shown that production of colicin V is not necessary for pathogenicity of *E. coli* by itself but it is a marker for other properties associated with the presence of ColV plasmid [61].

The operon *etsABC*, a new ABC transport system described for the first time in APEC strains by Johnson *et al.* [15], is located in a region downstream of *iss* (Fig. 2). This region contains about 10.5 kb of homology with segments in avian plasmids pAPEC-O2-ColV [15] and pAPEC-O1-ColBM [19]. EtsB is very similar to MacB (59% identity), an ABC-type membrane protein and the first macrolide antibiotic-specific drug exporter identified in gram-negative bacteria. The EtsA protein is 47% similar to MacA, a peripheral membrane protein that belongs to the membrane fusion protein family [62]. The fact that this region is flanked by ISs (Fig. S2A) could facilitate its propagation among avian plasmids.

We previously sequenced the region of *tsh* [16]. In this study, the full sequence of pAPEC-1 showed that similar to pAPEC-O2-ColV, the *tsh* gene in pAPEC-1 is located downstream of the ColV operon (Fig. 2). The *tsh* gene was identified and characterized for the first time in APEC strain χ7122 [63] and was confirmed later in other APEC strains [64] as well in UPEC strains [65]. Tsh, the first serine protease autotransporters of the *Enterobacteriaceae* (SPATEs), is used as a model for the study of the secretion and function of the proteins from the same family [66]–[68]. The *tsh* gene encodes for a temperature-sensitive hemagglutinin Tsh [63], which plays a role in the first steps of APEC infection [16]. This *tsh* gene is located on ColV and non-ColV plasmids [15], [16], [19]. The ability of Tsh to adhere to red blood cells, hemoglobin, and the extra-cellular matrix proteins fibronectin and collagen IV [69] are predictive of possible other roles for Tsh in virulence, such as acquisition of iron from heme [70].

Another interesting feature of the pAPEC-1 sequence is the presence of a unique region spanning 6.3 kb that contains two putative virulence genes *hlyF* and *ompT*. Overall, this region in pAPEC-1 shows homology with sequences of two avian plasmids pAPEC-O1-ColBM [19] and pAPEC-O2-ColV [15]. This region is flanked by two ISs (Fig. S2B) that could play a role in their propagation among APEC strains. Notably, three ORFs upstream of *ompT* are common in these APEC plasmids and do not have homology with other sequences in the public data base. The two ORFs downstream of *hlyF* share homology with the two aforementioned avian plasmids and a recently sequenced plasmid p1658/97 from a human septicemic *E. coli* [71]. The new class of hemolysin designated *hlyF* has been identified in *E. coli* strains isolated from broilers [72], but its role in virulence is still unknown. OmpT is a member of the omptins family found in different gram-negative bacteria [73]; the involvement of these omptins in the pathogenicity of these bacteria has been demonstrated by many studies [74]. In fact, OmpT is frequently associated with isolates of *E. coli* derived from complicated urinary tract disease, where it seems to play a role in the cleavage of protamine, an antimicrobial excreted by epithelial cells of the urinary tract [75]. OmpT is a protease VII that is thought to play a central role in protection against entry of foreign agents such as protenecious toxins into the cell [75], [76]. OmpT also catalyzes the activation of plasminigen to plasmin, a function associated with the virulence of *Yersinia pestis* and clinical *E. coli* isolates [77], [78]. The implication of the genes *ompT* and *hlyF* in the virulence of APEC should be investigated to better understand the pathogenicity of these strains.

### Insertion sequences

For the first time, we fully analyzed insertion sequences identified in an *E. coli* plasmid. Thirty three ORFs in pAPEC-1 are identified as insertion sequences (ISs). They belong to nine families including IS*1*, IS*3*, IS*4*, IS*21*, IS*66*, IS*91*, IS*110*, and ISNCy with diverse origins (Table S4). Three sequences of ISs (MM1_0049, MM1_0154, and MM_0155) in pAPEC-1 have no homology with sequences either in pAPEC-O2-ColV or pAPEC-O1-ColBM.

Insertion sequences (ISs) are involved in assembling genes into complex plasmid structures [79], [80]. Plasmids capable of self-transfer between strains have the potential to acquire insertion sequences with their neighboring genes. The ISs in pAPEC-1 may have mediated many transpositions events and segment acquisitions from different bacterial strains (Table S4), as indicated by the fluctuation of G+C content along the sequence of pAPEC-1.

### Comparative genomics

**Figure 5:**
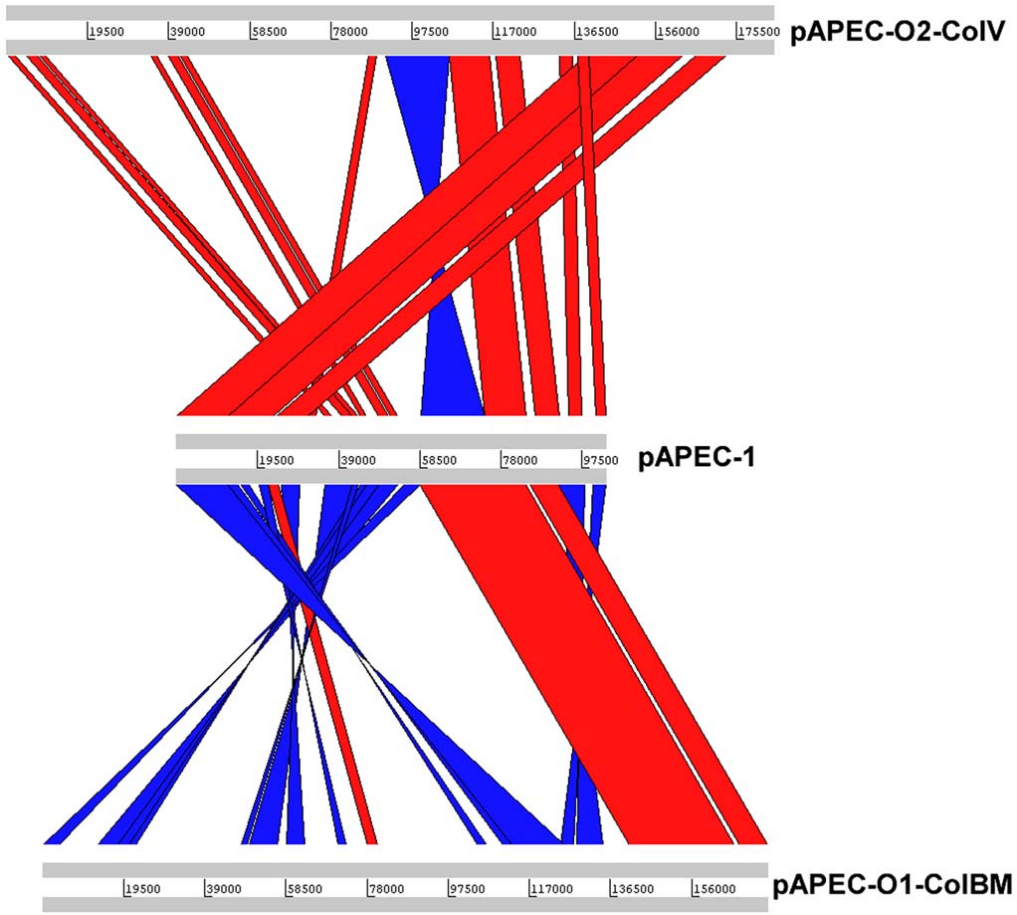
Pairwise nucleotide comparison of the complete pAPEC-1 DNA sequence to that of pAPEC-O1-ColBM (NC_009837) and pAPEC-O2-ColV (NC_007675). Same-strand DNA similarity is shaded red, while reverse similarity is shaded blue. Aligned regions greater than 1000 base pairs with a percent-identity >96% are shown. Comparisons were generated with the Artemis comparison tool, ACT [84].

We compared the pAPEC-1 plasmid with two other sequenced plasmids from APEC, pAPEC-1-O2-ColV [15] and pAPEC-O1-ColBM [19]. The three way comparison revealed that the three plasmids shared many blocs of DNA (Fig. 5). Compared to pAPEC-O2-ColV, the major deviations are the inversion of the region containing *sitABCD* and *iutA iucABCD* and the relocation of the region, including *iroBCDN*, *cvaABC*, and *tsh* located downstream of *iutA iucABCD* in pAPEC-1. Compared to pAPEC-O1-ColBM, the region downstream of *iutA iucABCD* in pAPEC-1 is inverted and relocated.

### Prevalence of pAPEC-1-related genes and their association in human ExPEC

pAPEC-1 associated virulence genes are more prevalent among APEC strains than non-APEC strains [15]. Growing evidence suggests that chickens are a suspected source of ExPEC in humans [81]. Therefore, we assessed the presence of 9 virulence genes of pAPEC-1, including *tsh*, *iss*, *cvaC*, *iroN*, *iucC*, *sitA*, *hlyF*, *ompT*, and *etsA*, by PCR among 100 human *E. coli* strains isolated from the main clinical extra-intestinal sources (UTI and non-UTI). Our results show that all genes tested were prevalent at different percentages in human ExPEC. Generally, pAPEC-1-associated virulence genes are more prevalent among non-UTI than UTI human isolates (Table 2). The most prevalent genes are those associated with the iron-acquisition systems, including *iroN* (52%), *iucC* (54%), and *sitA* (90%). Our analysis revealed many patterns of association between genes in human ExPEC (Table 3). This association includes fifteen percent that contains the *cvaC* gene, an indicator of the presence of the ColV plasmid and five percent that has the pattern *tsh-iss-cvaC-iroN-iucC-sitA-hlyF-ompT-etsA* similar of the one of pAPEC-1. All together, our results confirm the diversity of ExPEC strains and showed that human and avian *E. coli* can carry the same virulence plasmids, indicating the zoonotic risk of APEC strains. These common genes could be considered as potential candidates for a vaccine against a very diverse group of ExPEC either in human or chickens.

**Table 2:** Prevalence (%) of pAPEC-1 virulence-associated genes in human isolates.

Gene	UTI* (n = 50)	Non-UTI (n = 50)	Total (n = 100)
*tsh*	0	12	6
*iss*	8	26	17
*cvaC*	6	24	15
*iroN*	46	52	49
*iucC*	64	54	59
*sitA*	92	90	91
*hylF*	8	26	17
*ompT*	6	30	18
*etsA*	6	22	14

* UTI, Urinary Tract Infection.

**Table 3:** Prevalence of different pAPEC-1 gene combinations in human isolates.

No of Genes	% of positive isolates UTI (n = 50)	Pathotype (s)	% of positive isolate s Non-UTI (n = 50)	Pathotype (s)	Total (n = 100)
0	4	-	10	-	7
1	10	*iucC*	10	*sitA*	10
		*sitA*			
2	62	*iroN-sitA*	42	*iroN-sitA*	52
		*iucC-sitA*		*iucC-sitA*	
3	16	*iroN-iucC-sitA*	12	*iroN-iucC-sitA*	14
		*iroN-sitA-hlyF*		*iucC-sitA-ompT*	
				*iroN-sitA-ompT*	
				*iss-iucC-sitA*	
				*iss-iroN-sitA*	
4	2	*iss-iroN-iucC-sitA*	2	*iron-sitA-hlyF-ompT*	2
5	0	-	0	-	0
6	0	-	2	*iss-cvaC-iroN-sitA-hlyF-ompT*	1
7	2	*iss-cvaC-iroN-sitA-hlyF-ompT-etsA*	0	-	1
8	4	*iss-cvaC-iroN-iucC-sitA-hlyF-ompT-etsA*	12	*iss-cvaC-iroN-iucC-sitA-hlyF-ompT-etsA*	8
				*tsh-cvaC-iroN-iucC-sitA-hlyF-ompT-etsA*	
9	0	-	10	*tsh-iss-cvaC-iroN-iucC-sitA-hlyF-ompT-etsA*	5

* UTI, Urinary Tract Infection.

### pAPEC-1-associated virulence genes are conserved among some ExPEC strains

**Figure 6:**
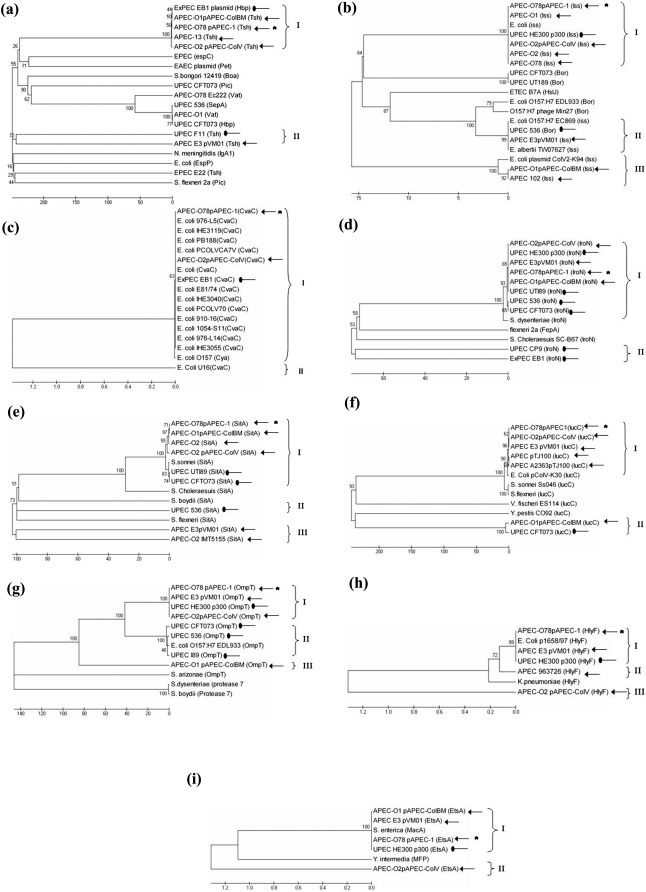
Phylogenetic relationships between protein sequences of nine pAPEC-1-associated virulence genes (a) *tsh*, (b) *iss*, (c) *cvaC*, (d) *iroN*, (e) *sitA*, (f) *iucC*, (g) *ompT*, (h) *hlyF*, and (i) *etsA* and related genes from other sources. The phylogenetic trees were constructed by neighbor-joining analysis. The phylogenetic groups are given on the right. Genes from pAPEC-1 (*); genes of APEC (normal arrow); and genes of other ExPEC (round arrow) are indicated.

The amino sequences of nine pAPEC-1-associated virulence genes (*tsh*, *iss*, *cvaC*, *iroN*, *iucC*, *sitA*, *hlyF*, *ompT* and *etsA*) were used to generate phylogenetic trees by the neighbor-joining method (Fig. 6 a–i). The same distinct clusters of strains were consistently grouped with high bootstrap values.

The phylogenetic trees show that pAPEC-1-associated virulence genes belong to clusters that include most genes of other ExPEC strains. The cluster with *tsh* contains other autotransporters (SPATEs) reported in *Enterobacteriaceae* (*vat*, *hbp*, *sepA*, and *pic*) [68] (Fig. 6 a). The gene annotated *hbp* in human pathogenic *E. coli* EB1 [82] was placed in the same cluster group of *tsh* (Fig. 6 a), which could be a discrepancy in annotation of the *hbp* gene. The large majority of *cvaC* genes from *E. coli* belong to the same large cluster (Fig. 6 c), which demonstrates that it is highly conserved among *E. coli* strains. The phylogenetic trees also show that the few sequences of *hlyF* and *etsA* available in GenBank are closely related (Fig. 6 h, i).

Analysis of the pAPEC-1 DNA sequence reveals that this ColV plasmid from an APEC strain O78∶K80∶H9 is a mosaic plasmid that is composed of a diverse set of genetic elements that may be acquired by transposition events due to flanking insertion sequences. The nature and the amount of DNA acquired depends on the pressure of selection imposed by the environment in which bacteria live and also the nature of neighboring flora from where they acquire new genes. The presence of two intact replication regions, RepFIB and RepFIIA, as well two different toxin-antitoxin systems, is evidence of the highly mosaic plasmid structure of pAPEC-1. The absence of some *tra* genes in pAPEC-1 affected its self-conjugative function and rendered it dependant on the machinery of other plasmids.

The comparison of pAPEC-1 sequence with the two available sequences of avian plasmids (pAPEC-O1-ColBM and pAPEC-O2-ColV) shows that the colicin V plasmids are not necessarily from the same backbone; in fact the regions encoding the functional gene products of pAPEC-1 share more similarity with the plasmid pAPEC-O1-ColBM than with plasmid pAPEC-O2-ColV.

pAPEC-1-associated virulence genes, especially those involved in iron-acquisition, are prevalent in human ExPEC. Different associations of individual genes are present in both UTI and non-UTI human isolates. The pathotype typical of pAPEC-1 is also found in some human strains, which indicates the zoonotic risk of APEC strains. The close phylogenic relationship of pAPEC-1-associated virulence genes with those of other ExPEC including Human strains supports this notion.

## Supporting Information

Table S1 List of primers used in this study. In this table, we present details of primers used in this study to determine the prevalence of pAPEC-1-associated virulence genes in Human ExPEC by PCR.
(0.03 MB DOC)

Table S2 Summary of information about the coding sequences of pAPEC-1. In this table, we present details of all coding sequences found in pAPEC-1
(0.33 MB DOC)

Table S3 ORFs involved in pAPEC-1 plasmid functions. In this table, we present all ORFs involved in pAPEC-1 plasmid functions, including replication, partition and stability.
(0.08 MB DOC)

Table S4 Insertion sequences identified in pAPEC-1. In this table, we present details of all insertion sequences identified in pAPEC-1
(0.08 MB DOC)

Figure S1 The genetic map of the replication and stability regions in pAPEC-1. This figure shows the genetic map of the replication and stability regions in pAPEC-1: The region of the stability containing the *sopABC* (A) and the second replicon *repFIC* region (B). Black arrows represent known ORFs genes, grey arrows represent truncated genes, white arrows represent hypothetical protein genes, and hatched arrows are Insertion Sequences genes.
(1.14 MB TIF)

Figure S2 The genetic map of the region of pAPEC-1 containing the *etsABC* (A) and *hlyF* and *ompT* genes (B). This figure shows the genetic map of the region of pAPEC-1 containing the *etsABC* (A) and *hlyF* and *ompT* genes (B). Black arrows represent known ORF genes, white arrows represent hypothetical protein genes, and hatched arrows are Insertion sequences genes.
(0.91 MB TIF)
