# Supplementary material for: Full Sequence and Comparative Analysis of the Plasmid pAPEC-1 of Avian Pathogenic E. coli χ7122 (O78∶K80∶H9)
Source: PLoS One. 2009 Jan 21;4(1):e4232. doi: 10.1371/journal.pone.0004232 (PMC2626276; doi:10.1371/journal.pone.0004232)
Supplement: Table S1 — List of primers used in this study. In this table, we present details of primers used in this study to determine the prevalence of pAPEC-1-associated virulence genes in Human ExPEC by PCR. (0.03 MB DOC) [file pone.0004232.s001.doc]

**Table S1.** List of primers used in this study.

| **genes** | **Primer sequence (5`-3`)** | **Amplicon size (bp)** |
| --- | --- | --- |
| *tsh* | F: GTTCAGGTCTGGTTTTTG  R: TCGCCCTTAACACCATT | 547 |
| *iss* | F: CCGAACCACTTGATGTGCA  R: CTATGCAAAAACAACTGTAG | 651 |
| *cvaC* | F: GGTATCCCTTCGGGTTTTTG  R: TGTTTCTGGTGGTGCTTCAG | 204 |
| *iroN* | F: ATTGACGCCAGGCATTTTAC  R: GCTCCTGGTTGGGTTGAATA | 202 |
| *iucC* | F: GACGGGCTTTCAGTAGTTGC  R: CTTCATCGCTGAACGTGGTA | 200 |
| *sitA* | F: ATCGGCATTACGTTGGTAGG  R: TCTCAATGGGGTTCCAGAAG | 196 |
| *hlyF* | F: TTAGATCCCCAGGCAAGATG  R: GGTGCAACAGGATTTCTTGG | 199 |
| *ompT* | F: CCTCCACGACCAGCTAATGT  R: CGGAGATTGATTTTGGCACT | 196 |
| *etsA* | F: GGATGCGGAAAGAACAGGTA  R: TTCTTCACTGGCATGGACTG | 203 |
