# Supplementary material for: Full Sequence and Comparative Analysis of the Plasmid pAPEC-1 of Avian Pathogenic E. coli χ7122 (O78∶K80∶H9)
Source: PLoS One. 2009 Jan 21;4(1):e4232. doi: 10.1371/journal.pone.0004232 (PMC2626276; doi:10.1371/journal.pone.0004232)
Supplement: Table S2 — Summary of information about the coding sequences of pAPEC-1. In this table, we present details of all coding sequences found in pAPEC-1 (0.33 MB DOC) [file pone.0004232.s002.doc]

**Table S2**. Summary of information about the coding sequences of pAPEC-1

| **GI Number** | **Position (bp)** | **Size (AA)** | **Gene symbol** | **Gene function** | **Expect Score** | **% identity** | **GeneBank reference** |
| --- | --- | --- | --- | --- | --- | --- | --- |
| MM1_0001 | 44-334 | 96 |  | CHP | 2.00e-50 | 98% | ZP_02903790 |
| MM1_0002 | 340-462 | 40 |  | HP |  |  |  |
| MM1_0003 | 444-1079 | 211 |  | Transposase | 1.00e-25 | 94% | [DQ388534](http://www.ebi.ac.uk/cgi-bin/emblfetch?DQ388534) |
| MM1_0004 | 1042-1161 | 39 |  | Transposase | 3.00e-31 | 93% | [DQ388534](http://www.ebi.ac.uk/cgi-bin/emblfetch?DQ388534) |
| MM1_0005 | 1466-1600 | 44 |  | CHP | 1.00e-07 | 71% | NP_753169 |
| MM1_0006 | 1646-1774 | 42 |  | CHP | 9.00e-08 | 76% | YP_668241 |
| MM1_0008 | 1847-2116 | 89 |  | HP |  |  |  |
| MM1_0007 | 2113-2262 | 49 |  | HP |  |  |  |
| MM1_0009 | 2315-3430 | 371 | *iroB* | Glycosyltransferase | 0 | 100% | YP_001481297 |
| MM1_0010 | 3444-7229 | 1261 | *iroC* | ABC transport protein | 0 | 99% | YP_444107 |
| MM1_0011 | 7333-8562 | 409 | *iroD* | Ferric enterochelin esterase | 0 | 99% | YP_444108 |
| MM1_0013 | 8647-9603 | 318 | *iroE* | Hydrolase | 0 | 99% | YP_444109 |
| MM1_0014 | 9648-11825 | 725 | *iroN* | Siderophore receptor | 0 | 100% | YP_001481293 |
| MM1_0015 | 12691-13725 | 344 | *aroH* | Aldolase | 2.00e-171 | 100% | YP_001481290 |
| MM1_0016 | 13743-13934 | 63 |  | HP |  |  |  |
| MM1_0017 | 13925-14070 | 48 |  | HP |  |  |  |
| MM1_0018 | 14285-14593 | 102 |  | CHP | 4.00e-51 | 100% | YP_001481287 |
| MM1_0019 | 14692-14874 | 60 |  | CHP | 3.00e-26 | 100% | YP_002039017 |
| MM1_0020 | 14871-15068 | 65 |  | CHP | 2.00e-30 | 100% | YP_443944 |
| MM1_0022 | 15220-15342 | 40 |  | HP |  |  |  |
| MM1_0021 | 15328-15444 | 38 |  | HP |  |  |  |
| MM1_0023 | 15588-15746 | 52 |  | HP |  |  |  |
| MM1_0024 | 15750-17024 | 424 | *cvaA* | Colicin V secretion | 0 | 99% | YP_001481279 |
| MM1_0025 | 17017-19113 | 698 | *cvaB* | Colicin V secretion | 0 | 100% | YP_444119 |
| MM1_0026 | 19283-19594 | 103 | *cvaC* | Colicin V synthesis | 4.00e-52 | 100% | YP_444120 |
| MM1_0027 | 19572-19808 | 78 | *cvi* | Colicin V immunity | 2.00e-36 | 100% | YP_444121 |
| MM1_0028 | 19690-19896 | 68 |  | HP |  |  |  |
| MM1_0029 | 19999-20370 | 123 |  | CHP | 1.00e-61 | 98% | YP_001481239 |
| MM1_0030 | 20342-20746 | 134 |  | CHP | 1.00e-73 | 100% | CAA11512 |
| MM1_0031 | 20733-21017 | 94 |  | CHP | 1.00e-16 | 51% | ZP_00834393 |
| MM1_0032 | 21014-21994 | 326 |  | Dimethylalanine monooxygenase | 0 | 97% | ABD51652 |
| MM1_0033 | 22061-22450 | 129 |  | Transposase | 0 | 99% | [NC_004431](http://www.ebi.ac.uk/cgi-bin/emblfetch?NC_004431) |
| MM1_0034 | 22447-22794 | 115 |  | Transposase | 0 | 99% | [NC_004431](http://www.ebi.ac.uk/cgi-bin/emblfetch?NC_004431) |
| MM1_0036 | 22844-24250 | 268 |  | Transposase | 0 | 99% | [NC_004431](http://www.ebi.ac.uk/cgi-bin/emblfetch?NC_004431) |
| MM1_0035 | 24235-24384 | 49 |  | HP |  |  |  |
| MM1_0038 | 24430-24687 | 85 |  | Resolvase | 2.5 | 100% | AF074611 |
| MM1_0037 | 24674-25126 | 150 | *def* | Peptide deformylase | 1.00e-77 | 98% | YP_444131 |
| MM1_0039 | 25235-29368 | 1377 | *tsh* | Adhesin/protease | 0 | 99% | YP_001481228 |
| MM1_0041 | 29491-29913 | 140 |  | CHP | 5.00e-78 | 100% | AAF76757 |
| MM1_0042 | 29973-30377 | 134 |  | Transposase | 1.00e-157 | 97% | X17613 |
| MM1_0043 | 30334-31464 | 376 |  | Transposase | 0 | 99% | [X62680](http://www.ebi.ac.uk/cgi-bin/emblfetch?X62680) |
| MM1_0044 | 31508-32287 | 259 |  | Transposase | 0 | 99% | [U59875](http://www.ebi.ac.uk/cgi-bin/emblfetch?U59875) |
| MM1_0045 | 32287-33309 | 340 |  | Transposase | 0 | 99% | [U59875](http://www.ebi.ac.uk/cgi-bin/emblfetch?U59875) |
| MM1_0046 | 33566-33931 | 121 |  | Transposase | 1.00e-144 | 93% | [X77671](http://www.ebi.ac.uk/cgi-bin/emblfetch?X77671) |
| MM1_0047 | 33886-34164 | 92 |  | Transposase | 1.00e-123 | 95% | [X17114](http://www.ebi.ac.uk/cgi-bin/emblfetch?X17114) |
| MM1_0049 | 34161-35015 | 284 |  | Transposase | 0 | 98% | [X17114](http://www.ebi.ac.uk/cgi-bin/emblfetch?X17114) |
| MM1_0048 | 34983-35129 | 48 |  | HP |  |  |  |
| MM1_0050 | 35227-35541 | 104 |  | HP |  |  |  |
| MM1_0051 | 35655-35933 | - | *repA4* | (truncated) |  |  |  |
| MM1_0052 | 36507-37364 | 285 | *repA1* | Replication protein | 5.00e-164 | 100% | YP_001481222 |
| MM1_0054 | 37357-37851 | 164 | *repA* | RepFIC initiation protein | 1.00e-91 | 99% | YP_001481221 |
| MM1_0169 | 37819-38121 | 100 | *repA3* | Replication protein | 4.00e-52 | 100% | YP_001481220 |
| MM1_0055 | 38138-38395 | 85 | *repA2* | Replication regulatory protein | 4.00e-41 | 100% | YP_001481219 |
| MM1_0056 | 38362-38529 | 55 |  | HP |  |  |  |
| MM1_0058 | 38575-38697 | 40 |  | HP |  |  |  |
| MM1_0057 | 38679-38990 | 103 | *srnB* | Post-segregation killing | 6.00e-53 | 99% | YP_001481218 |
| MM1_0059 | 38884-39093 | 69 |  | HP |  |  |  |
| MM1_0060 | 39159-39275 | 38 |  | Endonuclease | 6.00e-08 | 86% | YP_443951 |
| MM1_0061 | 39509-39721 | 70 |  | CHP | 7.00e-32 | 100% | YP_001481217 |
| MM1_0062 | 39857-40417 | 186 | *finO* | Fertility inhibition protein | 6.00e-103 | 100% | YP_001481216 |
| MM1_0063 | 40520-41380 | 286 |  | CHP | 3.00e-168 | 100% | YP_001481215 |
| MM1_0064 | 41439-42185 | 248 | *traX* | F pillin acetylation | 8.00e-140 | 100% | YP_001481214 |
| MM1_0065 | 42205-44088 | 627 | *traI* | DNA helicase I (truncated) | 0 | 97% | YP_001481213 |
| MM1_0066 | 44116-44275 | 73 | *traB* | Conjugal transfer protein (truncated) | 4.00e-22 | 100% | YP_001481188 |
| MM1_0067 | 44275-45003 | 242 | *traK* | Conjugal transfer | 1.00e-136 | 100% | NP_061456 |
| MM1_0068 | 44990-45556 | 188 | *traE* | F pilus assembly | 2.00e-106 | 100% | YP_001481186 |
| MM1_0069 | 45578-45889 | 103 | *traL* | F fimbriae outer membrane protein | 9.00e-54 | 100% | NP_061454 |
| MM1_0070 | 45904-46269 | 121 | *traA* | pilin protein | 3.00e-60 | 100% | YP_001481184 |
| MM1_0071 | 46302-46703 | 143 | *traY* | Conjugal transfer protein | 7.00e-71 | 98% | YP_001481183 |
| MM1_0072 | 46796-47485 | 229 | *traJ* | Positive regulator of conjugative transfer | 2.00e-130 | 99% | YP_001481182 |
| MM1_0073 | 47672-48055 | 127 | *traM* | DNA transfer | 1.00e-67 | 100% | YP_001481181 |
| MM1_0074 | 48388-48978 | 196 |  | Transglycosylase | 6.00e-112 | 98% | YP_001481180 |
| MM1_0075 | 49004-49138 | 44 |  | HP |  |  |  |
| MM1_0076 | 49157-49306 | 49 |  | HP |  |  |  |
| MM1_0077 | 49275-50204 | 309 |  | CHP | 5.00e-180 | 99% | YP_001481179 |
| MM1_0078 | 50274-50546 | 90 |  | HP |  |  |  |
| MM1_0079 | 50590-50793 | 67 |  | HP |  |  |  |
| MM1_0080 | 50902-51135 | 77 |  | CHP | 2.00e-35 | 98% | YP_190153 |
| MM1_0081 | 51142-51297 | 51 |  | HP |  |  |  |
| MM1_0082 | 51422-51538 | 38 | *hok* | Post-segregation killing | 2.00e-13 | 100% | NP_052939 |
| MM1_0168 | 51426-51638 | 70 | *mok* | Modulator of post-segregation killing | 6.00e-33 | 100% | NP_052938 |
| MM1_0083 | 51624-51848 | 74 | *sok* | Antisens RNA regulator | 3.00e-36 | 100% | YP_001481174 |
| MM1_0084 | 51860-52579 | 239 | *psiA* | Plasmid SOS inhibition protein A | 3.00e-132 | 97% | YP_190155 |
| MM1_0085 | 52576-53013 | 145 | *psiB* | Plasmid SOS inhibition protein B | 9.00e-79 | 98% | ZP_03051160 |
| MM1_0086 | 53065-53199 | 44 |  | CHP | 4.00e-19 | 100% | ABE10670 |
| MM1_0087 | 53290-53529 | 79 |  | CHP | 1.00e-39 | 100% | NP_061440 |
| MM1_0088 | 53591-54130 | 179 | *ssb* | Single-stranded DNA-binding protein | 6.00e-97 | 98% | YP_001481216 |
| MM1_0089 | 54156-54362 | 68 |  | HP |  |  |  |
| MM1_0090 | 54364-54918 | 184 |  | CHP | 8.00e-66 | 100% | YP_001739959 |
| MM1_0091 | 55458-56429 | 323 | *sopB* | Plasmid partition protein B | 0 | 100% | YP_001481159 |
| MM1_0092 | 56429-57604 | 391 | *sopA* | Plasmid partition protein A | 0 | 100% | YP_001481158 |
| MM1_0093 | 57895-58398 | 167 | *insB* | Transposon | 0 | 99% | [X52534](http://www.ebi.ac.uk/cgi-bin/emblfetch?X52534) |
| MM1_0094 | 58409-58621 | 70 |  | CHP | 9.00e-32 | 98% | YP_053131 |
| MM1_0095 | 58663-58968 | 101 |  | CHP | 6.00e-44 | 96% | YP_001481323 |
| MM1_0096 | 59077-61275 | 732 | *iutA* | Ferric aerobactin receptor | 0 | 99% | YP_001481324 |
| MM1_0097 | 61360-62637 | 425 | *iucD* | L-lysine 6-monooxygenase | 0 | 100% | P11295 |
| MM1_0098 | 62634-64376 | 580 | *iucC* | Aerobactin biosynthesis | 0 | 100% | YP_444060 |
| MM1_0099 | 64376-65323 | 315 | *iucB* | Aerobactin biosynthesis | 0 | 99% | YP_444059 |
| MM1_0100 | 65324-67105 | 593 | *iucA* | Aerobactin biosynthesis | 0 | 100% | YP_001481328 |
| MM1_0101 | 67020-67139 | 39 |  | HP |  |  |  |
| MM1_0102 | 67184-68377 | 297 | *shiF* | Transposase | 0.15 | 100% | AF060182 |
| MM1_0103 | 68493-68609 | 38 |  | HP |  |  |  |
| MM1_0104 | 68757-69137 | 126 | *crcB* | Chromosome condensation | 2.00e-63 | 100% | YP_001481330 |
| MM1_0105 | 69478-69912 | 129 | *eno* | Enolase | 9.00e-58 | 99% | YP_001481332 |
| MM1_0106 | 70268-70414 | 48 |  | HP |  |  |  |
| MM1_0107 | 70380-71237 | 285 | *sitD* | Chelated iron transport system | 2.00e-157 | 100% | YP_001481333 |
| MM1_0108 | 71234-72091 | 285 | *sitC* | Chelated iron transport system | 2.00e-158 | 100% | ABD51749 |
| MM1_0109 | 72088-72915 | 275 | *sitB* | Iron transport protein | 8.00e-158 | 100% | ABA54732 |
| MM1_0110 | 72915-73829 | 304 | *sitA* | Periplasmic chelator iron-binding | 9.00e-179 | 100% | YP_001481336 |
| MM1_0111 | 74185-74460 | 91 | *insA* | Transposase | 1.00e-152 | 99% | [J01730 [V]](http://www.ebi.ac.uk/cgi-bin/emblfetch?J01730 %5BV%5D) |
| MM1_0112 | 74379-74882 | 167 |  | Transposase | 0 | 99% | [X52534](http://www.ebi.ac.uk/cgi-bin/emblfetch?X52534) |
| MM1_0113 | 74911-75060 | 58 | *parB* | Plasmid stability protein (truncated) |  |  |  |
| MM1_0114 | 75062-75451 | 129 | *impB* | UV protection and mutation | 1.00e-67 | 99% | BAA75113 |
| MM1_0115 | 75709-76065 | 118 | *istA* | Transposase | 1.00e-178 | 99% | [X14793](http://www.ebi.ac.uk/cgi-bin/emblfetch?X14793) |
| MM1_0116 | 76287-76511 | 74 |  | HP |  |  |  |
| MM1_0117 | 76648-76818 | 118 |  | HP |  |  |  |
| MM1_0118 | 76811-77788 | 325 | *repB* | RepFIB replication protein B | 2e-170 | 99% | YP_001481342 |
| MM1_0119 | 77864-77983 | 39 |  | HP |  |  |  |
| MM1_0120 | 78073-78813 | 246 | *intM* | Transposase | 1.4 | 94% | [NC_007907](http://www.ebi.ac.uk/cgi-bin/emblfetch?NC_007907) |
| MM1_0121 | 78970-79095 | 41 |  | HP |  |  |  |
| MM1_0122 | 79496-80404 |  |  | CHP | 2.00e-177 | 100% | YP_001481344 |
| MM1_0123 | 80467-81576 | 369 | *hylF* | Hemolysin F | 0 | 100% | YP_001481345 |
| MM1_0124 | 81826-81981 | 51 |  | HP |  |  |  |
| MM1_0125 | 82009-82962 | 317 | *ompT* | Outer membrane protease | 0 | 100% | YP_444072 |
| MM1_0126 | 83066-83455 | 129 |  | CHP | 6.00e-69 | 99% | YP_444073 |
| MM1_0127 | 83594-83731 | 45 |  | HP |  |  |  |
| MM1_0128 | 83823-84086 | 87 |  | HP |  |  |  |
| MM1_0129 | 84209-84457 | 83 |  | Transposase | 1.00e-14 | 82% | [Z83734](http://www.ebi.ac.uk/cgi-bin/emblfetch?Z83734) |
| MM1_0130 | 84577-84903 | 108 |  | Transposase | 1.00e-163 | 96% | [U06468](http://www.ebi.ac.uk/cgi-bin/emblfetch?U06468) |
| MM1_0131 | 84903-85094 | 63 |  | Transposase | 5.00e-96 | 99% | X51586 |
| MM1_0132 | 85078-85776 | 232 |  | Transposase | 0 | 96% | [X51586](http://www.ebi.ac.uk/cgi-bin/emblfetch?X51586) |
| MM1_0133 | 86046-86336 | 96 |  | Transposase | 4.00e-79 | 96% | [X17613](http://www.ebi.ac.uk/cgi-bin/emblfetch?X17613) |
| MM1_0134 | 86358-86486 | 42 |  | HP |  |  |  |
| MM1_0135 | 87096-87224 | 42 |  | HP |  |  |  |
| MM1_0136 | 87230-88417 | 317 | *etsA* | Macrolide-specific efflux protein EtsA | 0 | 99% | YP_001481352 |
| MM1_0137 | 88414-90354 | 395 | *etsB* | Macrolide export ATP-binding/permease | 0 | 99% | YP_444080 |
| MM1_0138 | 90358-91728 | 646 | *etsC* | Outer membrane protein | 0 | 100% | YP_444081 |
| MM1_0139 | 91731-91859 | 42 |  | HP |  |  |  |
| MM1_0140 | 91856-91999 | 47 |  | HP |  |  |  |
| MM1_0141 | 92057-92245 | 62 |  | CHP | 9.00e-24 | 91% | YP_001588353 |
| MM1_0142 | 92347-92502 | 51 |  | HP |  |  |  |
| MM1_0143 | 92525-93466 | 313 |  | CHP | 0 | 100% | YP_001481312 |
| MM1_0144 | 93476-93664 | 62 |  | HP |  |  |  |
| MM1_0145 | 93701-94087 | 128 |  | CHP | 2.00e-68 | 100% | YP_001481310 |
| MM1_0146 | 94224-95090 | 288 |  | Transposase | 0.11 | 100% |  |
| MM1_0147 | 95277-95588 | 103 |  | Transposase | 1.00e-57 | 93% | [M18426](http://www.ebi.ac.uk/cgi-bin/emblfetch?M18426) |
| MM1_0148 | 95605-95757 | 50 |  | HP |  |  |  |
| MM1_0149 | 95727-96920 | 397 |  | CHP | 0 | 100% | YP_444097 |
| MM1_0150 | 97335-97559 | 74 |  | HP |  |  |  |
| MM1_0151 | 97540-97659 | 39 |  | HP |  |  |  |
| MM1_0152 | 97809-97973 | 55 |  | Transposase | 5.00e-09 | 85% | [Z48244](http://www.ebi.ac.uk/cgi-bin/emblfetch?Z48244) |
| MM1_0153 | 98162-98902 | 247 |  | Transposase | 0 | 98% | [M18426](http://www.ebi.ac.uk/cgi-bin/emblfetch?M18426) |
| MM1_0154 | 98952-99251 | 99 |  | Transposase | 1.00e-171 | 100% | [DQ388534](http://www.ebi.ac.uk/cgi-bin/emblfetch?DQ388534) |
| MM1_0155 | 99248-100114 | 298 |  | Transposase | 0 | 99% | [DQ388534](http://www.ebi.ac.uk/cgi-bin/emblfetch?DQ388534) |
| MM1_0156 | 100128-100328 | 66 |  | Transposase | 1.00e-102 | 100% | [M18426](http://www.ebi.ac.uk/cgi-bin/emblfetch?M18426) |
| MM1_0157 | 100286-100651 | 121 |  | Transposase | 0 | 100% | [M18426](http://www.ebi.ac.uk/cgi-bin/emblfetch?M18426) |
| MM1_0158 | 100882-101004 | 40 |  | HP |  |  |  |
| MM1_0159 | 101005-101133 | 42 |  | HP |  |  |  |
| MM1_0160 | 101261-101569 | 102 | *iss* | Increased serum survival | 8.00e-53 | 100% | YP_444101 |
| MM1_0161 | 101582-101875 | 97 |  | CHP | 1.00e-47 | 100% | YP_001481301 |
| MM1_0162 | 101890-102015 | 41 |  | HP |  |  |  |
| MM1_0163 | 102085-102267 | 60 |  | HP |  |  |  |
| MM1_0164 | 102460-102732 | 90 |  | CHP | 8.00e-47 | 98% | ZP_02903790 |
| MM1_0165 | 102738-102860 | 40 |  | HP |  |  |  |
| MM1_0166 | 102842-103273 | 143 |  | Transposase | 0.2 | 100% | [NC_006393](http://www.ebi.ac.uk/cgi-bin/emblfetch?NC_006393) |

AA, amino acid; GI, GenInfo Identifier; AA, amino acid; Hp, hypothetical protein; CHP, conserved hypothetical protein.
