# Supplementary material for: Full Sequence and Comparative Analysis of the Plasmid pAPEC-1 of Avian Pathogenic E. coli χ7122 (O78∶K80∶H9)
Source: PLoS One. 2009 Jan 21;4(1):e4232. doi: 10.1371/journal.pone.0004232 (PMC2626276; doi:10.1371/journal.pone.0004232)
Supplement: Table S3 — ORFs involved in pAPEC-1 plasmid functions. In this table, we present all ORFs involved in pAPEC-1 plasmid functions, including replication, partition and stability. (0.08 MB DOC) [file pone.0004232.s003.doc]

**Table S3.** ORFs involved in pAPEC-1 plasmid functions

| **Gene Id** | **End5-end3** | **Number**  **aa** | **Gene symbol** | **function** | **GenBank match (accession no.)** | **% identity** | **p-value** |
| --- | --- | --- | --- | --- | --- | --- | --- |
| **replication** |  |  |  |  |  |  |  |
| MM1_0052 | 37364-36507 | 285 | *repA1* | Replication protein, IncFII RepA | YP_001481222 | 100 | 5.00e-164 |
| MM1_0054 | 37851-37357 | 164 | *repA* | RepFIC initiation protein | YP_001481221 | 99 | 1.00e-91 |
| MM1_0169 | 37819-38121 | 100 | *repA3* | Replication protein | YP_001481220 | 100 | 4.00e-52 |
| MM1_0055 | 38395-38138 | 85 | *repA2* | Replication regulatory protein | YP_001481219 | 100 | 4.00e-41 |
| MM1_0118 | 76811-77788 | 325 | *repB* | repFIB replication protein B | YP_001481342 | 99 | 2e-170 |
| **Transfer** |  |  |  |  |  |  |  |
| MM1_0064 | 42185-41439 | 248 | *traX* | F pillin acetylation protein | YP_001481214 | 100 | 8.00e-140 |
| MM1_0065 | 44088-42205 | 627 | *traI* | DNA helicase I (truncated) | YP_001481213 | 97 | 0 |
| MM1_0066 | 44116-44275 | 73 | *traB* | Conjugal transfer protein (truncated) | YP_001481188 | 100 | 4.00e-22 |
| MM1_0067 | 45003-44275 | 242 | *traK* | Conjugal transfer protein | NP_061456 | 100 | 1.00e-136 |
| MM1_0068 | 45556-44990 | 188 | *traE* | F pilus assembly protein | YP_001481186 | 100 | 2.00e-106 |
| MM1_0069 | 45889-45578 | 103 | *traL* | F fimbriae outer membrane protein | NP_061454 | 100 | 9.00e-54 |
| MM1_0070 | 46269-45904 | 121 | *traA* | pilin protein | YP_001481184 | 100 | 3.00e-60 |
| MM1_0071 | 46703-46302 | 143 | *traY* | Conjugal transfer protein | YP_001481183 | 98 | 7.00e-71 |
| MM1_0072 | 47485-46796 | 229 | *traJ* | Positive regulator of conjugative transfer | YP_001481182 | 99 | 2.00e-130 |
| MM1_0073 | 48055-47672 | 127 | *traM* | Essential part of the DNA transfer machinery | YP_001481181 | 100 | 1.00e-67 |
| **Partition/**  **stability** |  |  |  |  |  |  |  |
| MM_0057 | 38990-38679 | 103 | *srnB* | Post-segregation killing | YP_001481218 | 99 | 6.00e-53 |
| MM1_0091 | 56429-55458 | 323 | *sopB* | Plasmid partition protein B | YP_001481159 | 100 | 0 |
| MM1_0092 | 57604-56429 | 391 | *sopA* | Plasmid partition protein A | YP_001481158 | 100 | 0 |
| MM1_0113 | 74911-75060 | 58 | *parB* | Plasmid stability protein (truncated) |  |  |  |
| MM1_0082 | 51538-51422 | 38 | *hok* | Post-segregation killing | NP_052939 | 100 | 2.00e-13 |
| MM1_0168 | 51638-51426 | 70 | *mok* | Modulator of post-segregation killing | NP_052938 | 100 | 6.00e-33 |
| MM1_0083 | 51624-51848 | 74 | *sok* | Antisens RNA regulator | YP_001481174 | 100 | 3.00e-36 |
| **Others** |  |  |  |  |  |  |  |
| MM1_0062 | 40417-39857 | 186 | *finO* | Fertility inhibition protein | YP_001481216 | 100 | 6.00e-103 |
| MM1_0084 | 52579-51860 | 239 | *psiA* | Plasmid SOS inhibition protein A | YP_190155 | 97 | 3.00e-132 |
| MM1_0085 | 53013-52576 | 145 | *psiB* | Plasmid SOS inhibition protein B | ZP_03051160 | 98 | 9.00e-79 |
| MM1_0088 | 54130-53591 | 179 | *ssb* | Single-stranded DNA-binding protein | YP_001481216 | 98 | 6.00e-97 |
| MM1_0104 | 69137-68757 | 126 | *crcB* | Chromosome condensation | YP_001481330 | 100 | 2.00e-63 |
| MM1_0114 | 75451-75062 | 129 | *impB* | Involved in UV protection and mutation | BAA75113 | 99 | 1.00e-67 |
