# Supplementary material for: Full Sequence and Comparative Analysis of the Plasmid pAPEC-1 of Avian Pathogenic E. coli χ7122 (O78∶K80∶H9)
Source: PLoS One. 2009 Jan 21;4(1):e4232. doi: 10.1371/journal.pone.0004232 (PMC2626276; doi:10.1371/journal.pone.0004232)
Supplement: Table S4 — Insertion sequences identified in pAPEC-1. In this table, we present details of all insertion sequences identified in pAPEC-1 (0.08 MB DOC) [file pone.0004232.s004.doc]

**Table S4.**  Insertion sequences identified in pAPEC-1

| **Gene Id** | **End5-end3** | **Nb. aa** | **Sequences producing significant alignments** | **Family/**  **Group** | **origin** | **GenBank match (accession no.)** | **% identity** | **Score (bits)/E value** |
| --- | --- | --- | --- | --- | --- | --- | --- | --- |
| MM1_0003 | 444-1079 | 211 | ISEc*13* | IS*4*/IS*50* | [EPEC](http://www.ebi.ac.uk/cgi-bin/emblfetch?DQ388534) | [DQ388534](http://www.ebi.ac.uk/cgi-bin/emblfetch?DQ388534) | 94 | 113 (57)/1e-25 |
| MM1_0004 | 1042-1161 | 39 | ISEc*13* | IS*4*/IS*50* | EPEC | [DQ388534](http://www.ebi.ac.uk/cgi-bin/emblfetch?DQ388534) | 93 | 129 (65)/3e-31 |
| MM1_0033 | 22061-22450 | 129 | ISEc*8* | IS*66*/- | O157:H7 | [NC_004431](http://www.ebi.ac.uk/cgi-bin/emblfetch?NC_004431) | 99 | 751 (379)/0.0 |
| MM1_0034 | 22447-22794 | 115 | ISEc*8* | IS*66*/- | O157:H7 | [NC_004431](http://www.ebi.ac.uk/cgi-bin/emblfetch?NC_004431) | 99 | 666 (336)/0.0 |
| MM1_0036 | 22844-24250 | 268 | ISEc*8* | IS*66*/- | O157:H7 | [NC_004431](http://www.ebi.ac.uk/cgi-bin/emblfetch?NC_004431) | 99 | 2704 (1364)/0.0 |
| MM1_0038 | 24430-24687 | 85 | IS*1618* | IS*110*/IS*1111* | *Y. pestis* | AF074611 | 100 | 28.2(14)/1.9 |
| MM1_0042 | 29973-30377 | 134 | IS*911* | IS*3*/IS*3* | *S. dysenteriae* | X17613 | 97 | 551 (278)/e-158 |
| MM1_0043 | 30334-31464 | 376 | IS*30D* | IS*30*/- | *E. coli* K-12 | [X62680](http://www.ebi.ac.uk/cgi-bin/emblfetch?X62680) | 99 | 2173 (1096)/0.0 |
| MM1_0044 | 32287-31508 | 259 | IS*100kyp* | IS*21*/- | *Y.pseudotuberculosis* | [U59875](http://www.ebi.ac.uk/cgi-bin/emblfetch?U59875) | 99 | 1538 (776)/0.0 |
| MM1_0045 | 33309-32287 | 340 | IS*100kyp* | IS*21*/- | *Y.pseudotuberculosis* | [U59875](http://www.ebi.ac.uk/cgi-bin/emblfetch?U59875) | 99 | 2020 (1019)/0.0 |
| MM1_0046 | 33566-33931 | 121 | IS*91* | IS*91*/- | *E. coli* K88 | [X77671](http://www.ebi.ac.uk/cgi-bin/emblfetch?X77671) | 93 | 505 (255)/e-144 |
| MM1_0047 | 33886-34164 | 92 | IS*91* | IS*91*/- | *E. coli* EC185 | [X17114](http://www.ebi.ac.uk/cgi-bin/emblfetch?X17114) | 95 | 436 (220)/e-123 |
| MM1_0049 | 34161-35015 | 284 | IS*91* | IS*91*/- | *E. coli* EC185 | [X17114](http://www.ebi.ac.uk/cgi-bin/emblfetch?X17114) | 98 | 1566 (790)/0.0 |
| MM1_0093 | 58398-57895 | 167 | IS*1A* | IS*1*/- | *E. coli* W3110 | [X52534](http://www.ebi.ac.uk/cgi-bin/emblfetch?X52534) | 99 | 975 (492)/0.0 |
| MM1_0102 | 67184-68377 | 297 | IS*1601* | IS*256*/- | *M. avium* | AF060182 | 100 | 34.2 (17)/0.15 |
| MM1_0111 | 74185-74460 | 91 | IS*1G* | IS*1*/- | *E. coli* C600 | [J01730 [V]](http://www.ebi.ac.uk/cgi-bin/emblfetch?J01730 %5BV%5D) | 99 | 531/e-152 |
| MM1_0112 | 74379-74882 | 167 | IS*1A* | IS*1*/- | *E. coli* W3110 | [X52534](http://www.ebi.ac.uk/cgi-bin/emblfetch?X52534) | 99 | 983(496)/0.0 |
| MM1_0115 | 76065-75709 | 118 | IS*21* | IS*21*/- | *P. aerugenosa* | [X14793](http://www.ebi.ac.uk/cgi-bin/emblfetch?X14793) | 99 | 618 (312)/e-178 |
| MM1_0120 | 78813-78073 | 246 | IS*Dha7* | IS*3*/IS*3* | *D. hafniense* Y51 | [NC_007907](http://www.ebi.ac.uk/cgi-bin/emblfetch?NC_007907) | 94 | 30.2 (15)/1.4 |
| MM1_0129 | 84393-84235 | 53 | IS*1351* | IS*3*/IS*407* | *S. enteretedis* | [Z83734](http://www.ebi.ac.uk/cgi-bin/emblfetch?Z83734) | 82 | 75.8 (38)/5e-15 |
| MM1_0130 | 84577-84903 | 108 | IS*1203* | IS*3*/IS*51* | *E. coli* O111 | [U06468](http://www.ebi.ac.uk/cgi-bin/emblfetch?U06468) | 96 | 569(287)/e-163 |
| MM1_0131 | 84903-85094 | 63 | IS*629* | IS*3*/IS*51* | *S. sonnei* | X51586 | 99 | 345(174/5e-96 |
| MM1_0132 | 85078-85776 | 232 | IS*629* | IS*3*/IS*51* | *S. sonnei* | [X51586](http://www.ebi.ac.uk/cgi-bin/emblfetch?X51586) | 96 | 1205(608)/0.0 |
| MM1_0133 | 86336-86046 | 96 | IS*911* | IS*3*/IS*3* | *S. dysenteriae* | [X17613](http://www.ebi.ac.uk/cgi-bin/emblfetch?X17613) | 96 | 289 (146)/4e-79 |
| MM1_0146 | 95090-94224 | 288 | IS*Bce13* | IS*3*/IS*3* | *B. cereus* | - | 100 | 34.2(17)/0.11 |
| MM1_0147 | 95588-95277 | 103 | IS*2* | IS*3*/IS*2* | *E. coli K-12* | [M18426](http://www.ebi.ac.uk/cgi-bin/emblfetch?M18426) | 93 | 218 (110)/1e-57 |
| MM1_0152 | 97809-97973 | 55 | IS*1328* | IS*110*/IS*1111* | *Y. enterocolitica* | [Z48244](http://www.ebi.ac.uk/cgi-bin/emblfetch?Z48244) | 85 | 56.0(28)/5e-09 |
| MM1_0153 | 98902-98162 | 247 | IS*2* | IS*3*/IS*2* | *E. coli* | [M18426](http://www.ebi.ac.uk/cgi-bin/emblfetch?M18426) | 98 | 1334 (673)/0.0 |
| MM1_0154 | 98952-99251 | 99 | IS*Ec17* | IS*3*/IS*3* | *E. coli* | [DQ388534](http://www.ebi.ac.uk/cgi-bin/emblfetch?DQ388534) | 100 | 595 (300)/e-171 |
| MM1_0155 | 99248-100114 | 298 | IS*Ec17* | IS*3*/IS*3* | *E. coli* | [DQ388534](http://www.ebi.ac.uk/cgi-bin/emblfetch?DQ388534) | 99 | 1711 (863)/0.0 |
| MM1_0156 | 100328-100128 | 66 | IS*2* | IS*3*/IS*2* | *E. coli K12* | [M18426](http://www.ebi.ac.uk/cgi-bin/emblfetch?M18426) | 100 | 365 (184)/e-102 |
| MM1_0157 | 100651-100286 | 121 | IS*2* | IS*3*/IS*2* | *E. coli K12* | [M18426](http://www.ebi.ac.uk/cgi-bin/emblfetch?M18426) | 100 | 726 (366)/0.0 |
| MM1_0166 | 102842-103273 | 143 | IS*Hma5* | IS*NCy*/IS*H6* | *H. marismortui* | [NC_006393](http://www.ebi.ac.uk/cgi-bin/emblfetch?NC_006393) | 100 | 32.2 bits (16)/0.20 |
