# Supplementary figures and images for: Full Sequence and Comparative Analysis of the Plasmid pAPEC-1 of Avian Pathogenic E. coli χ7122 (O78∶K80∶H9)
Source: PLoS One. 2009 Jan 21;4(1):e4232. doi: 10.1371/journal.pone.0004232 (PMC2626276; doi:10.1371/journal.pone.0004232)

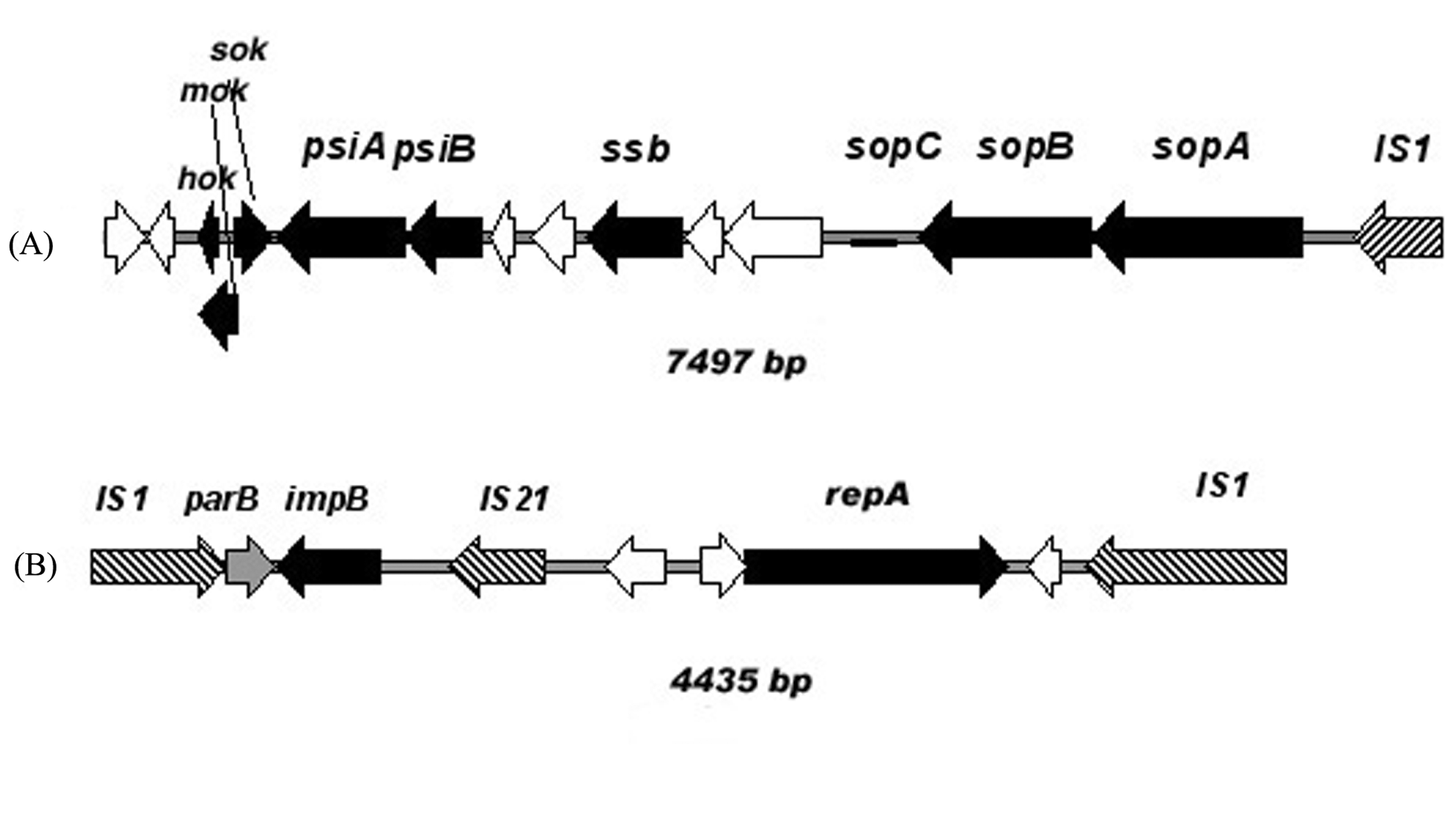

Supplement: Figure S1 — The genetic map of the replication and stability regions in pAPEC-1. This figure shows the genetic map of the replication and stability regions in pAPEC-1: The region of the stability containing the sopABC (A) and the second replicon repFIC region (B). Black arrows represent known ORFs genes, grey arrows represent truncated genes, white arrows represent hypothetical protein genes, and hatched arrows are Insertion Sequences genes. (1.14 MB TIF) [file pone.0004232.s005.tif]

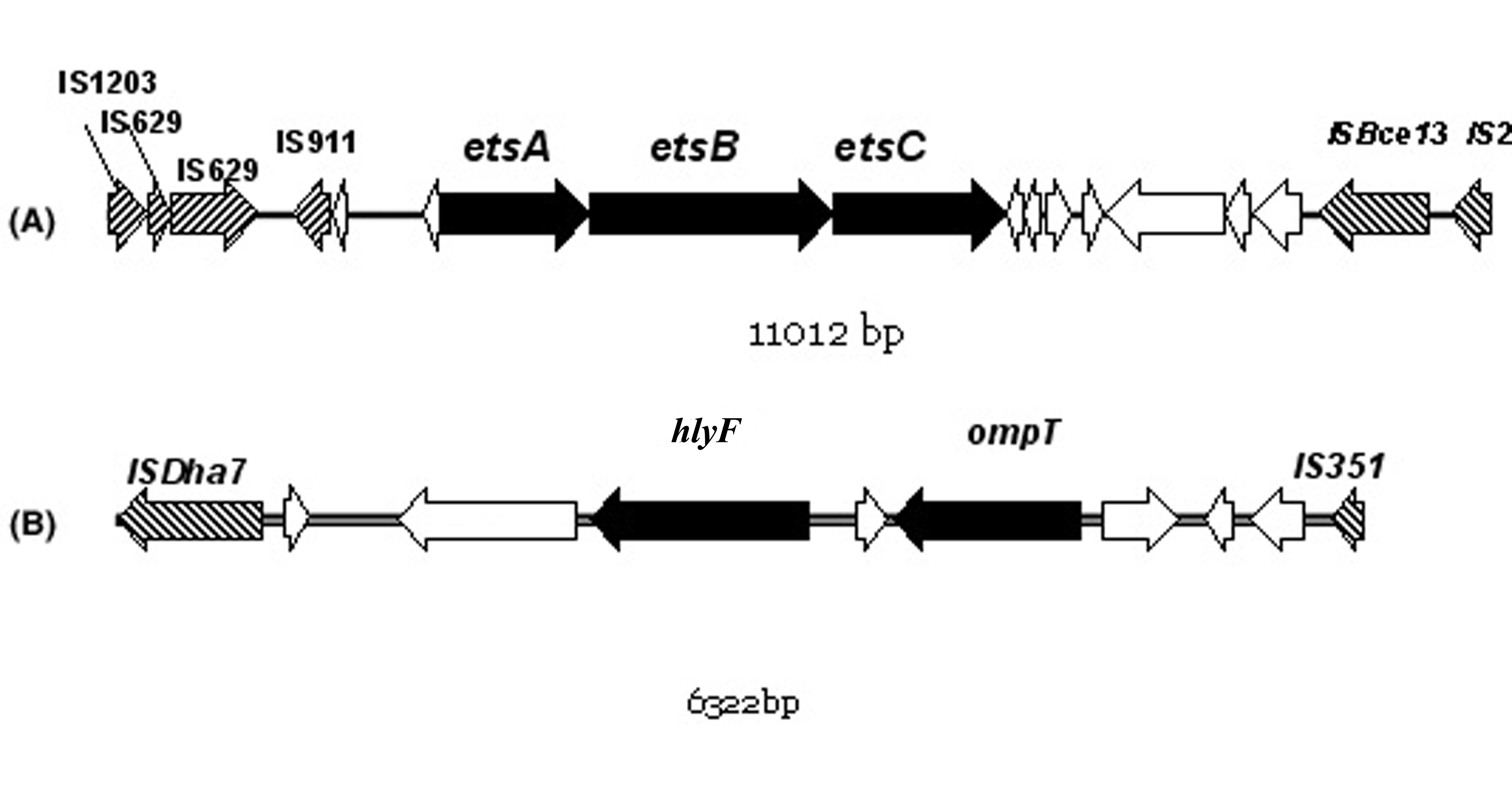

Supplement: Figure S2 — The genetic map of the region of pAPEC-1 containing the etsABC (A) and hlyF and ompT genes (B). This figure shows the genetic map of the region of pAPEC-1 containing the etsABC (A) and hlyF and ompT genes (B). Black arrows represent known ORF genes, white arrows represent hypothetical protein genes, and hatched arrows are Insertion sequences genes. (0.91 MB TIF) [file pone.0004232.s006.tif]
